# Construction and transformation of a *Thermotoga-E. coli *shuttle vector

**DOI:** 10.1186/1472-6750-12-2

**Published:** 2012-01-06

**Authors:** Dongmei Han, Stephen M Norris, Zhaohui Xu

**Affiliations:** 1 Department of Biological Sciences, Bowling Green State University, Bowling Green, OH 43403, USA; 2 Center for Photochemical Sciences, Bowling Green State University, Bowling Green, OH 43403, USA

## Abstract

**Background:**

*Thermotoga *spp. are attractive candidates for producing biohydrogen, green chemicals, and thermostable enzymes. They may also serve as model systems for understanding life sustainability under hyperthermophilic conditions. A lack of genetic tools has hampered the investigation and application of these organisms. This study aims to develop a genetic transfer system for *Thermotoga *spp.

**Results:**

Methods for preparing and handling *Thermotoga *solid cultures under aerobic conditions were optimized. A plating efficiency of ~50% was achieved when the bacterial cells were embedded in 0.3% Gelrite. A *Thermotoga-E. coli *shuttle vector pDH10 was constructed using pRQ7, a cryptic mini-plasmid found in *T*. sp. RQ7. Plasmid pDH10 was introduced to *T. maritima *and *T*. sp. RQ7 by electroporation and liposome-mediated transformation. Transformants were isolated, and the transformed kanamycin resistance gene (*kan*) was detected from the plasmid DNA extracts of the recombinant strains by PCR and was confirmed by restriction digestions. The transformed DNA was stably maintained in both *Thermotoga *and *E. coli *even without the selective pressure.

**Conclusions:**

*Thermotoga *are transformable by multiple means. Recombinant *Thermotoga *strains have been isolated for the first time. A heterologous *kan *gene is functionally expressed and stably maintained in *Thermotoga*.

## Background

Besides *Aquifex*, *Thermotoga *are the only group of bacteria that can grow up to 90°C. Isolates of *Thermotoga *have been discovered from heated sea floors [1], continental hot springs [2], and oil fields [3]. Analysis of their 16S rRNA sequences have positioned *Thermotoga *spp. to a deep branch of the tree of life, suggesting that these strict anaerobes emerged at an early stage of evolution, when the surface of the Earth was hot and its atmosphere contained little oxygen. Study of the molecular genetics of *Thermotoga *is expected to shed light on the fundamental questions related to the origin of life as well as the mechanisms of the thermostability of macromolecules under extreme conditions. Most importantly, *Thermotoga *hydrolyze a number of polysaccharides through fermentative catabolism and produce hydrogen gas as one of the final products [4]. This has stimulated tremendous interest in utilizing these bacteria to produce biomass-based clean energy, especially through metabolic engineering approaches. However, due to the lack of genetic tools, the investigations of *Thermotoga *are still largely limited to biochemical, genomic, and fermentative studies, as with most hyperthermophiles.

Cryptic mini-plasmids pRQ7, pMC24, and pRKU1 have been identified in *T*. sp. RQ7 [5], *T. maritima *[6], and *T. petrophila *RKU-1 [7], respectively. Although discovered at geologically unrelated locations, the three plasmids are nearly identical. They are extremely small (846 bp) and encode just one apparent open reading frame, presumably the replication protein. Studies of pRQ7 suggest that the plasmid is negatively supercoiled and replicates by a rolling-circle mechanism [5,8]. Based on pRQ7, two *Thermotoga*-*E. coli *shuttle vectors pJY1 (chloramphenicol-resistant) and pJY2 (kanamycin-resistant) have been constructed for expression in *T. neapolitana *and *T. maritima*, respectively [9]. Through liposome-mediated transformation, both vectors rendered transient antibiotic resistance to *Thermotoga *cells in liquid media, but no transformants could be isolated from plates. To date, that report remains the only documented effort of expressing heterologous genes in *Thermotoga*, out of more than 1200 publications retrieved from PubMed using "*Thermotoga*" as the key word (last searched December 20, 2011). In fact, genetic manipulation of *Thermotoga *remains a challenge. To develop a tractable gene transfer system for *Thermotoga *spp., we systematically examined every aspect pertaining to the cloning and expression of foreign genes in *Thermotoga*, from plating efficiency to vector stability. We demonstrate that heterologous genes can be introduced to *Thermotoga *through multiple means, be functionally expressed, and be stably maintained.

## Methods

### Strains and growth conditions

Bacterial strains and vectors involved in this study are listed in Table 1. *E. coli *DH5α was used as the host strain for the construction of vectors and was grown at 37°C in Luria Broth (1% tryptone, 0.5% NaCl, 0.5% yeast extract), with 1.5% agar for plates. Ampicillin was supplemented at 100 μg ml^-1 ^when needed. *Thermotoga neapolitana *ATCC 49049 (same as *T*. *neapolitana *DSM 4359) was purchased from ATCC http://www.atcc.org/, and strains *T*. sp. RQ7 and *T. maritima *MSB8 were kindly provided by Dr. Harald Huber, University of Regensburg, Germany. *Thermotoga *were cultivated at 77°C in SVO medium developed by van Ooteghem et al. [10]. Fifty milliliters of SVO was dispensed into 100 ml serum bottles and sparged with nitrogen gas to remove oxygen from the medium and the headspace. Serum bottles were then sealed by rubber stoppers, secured by aluminum caps, and sterilized. Inoculation of the liquid SVO was done by a syringe needle with a typical inoculum of 2%. Liquid cultures were shaken at 100 rpm. For preparation of soft SVO, 0.075% Gelrite (Sigma-Aldrich Co., St. Louis, MO, USA) was dissolved in liquid SVO. Culture tubes with screw caps were filled with soft SVO up to two thirds of the volume capacity and were autoclaved. To grow *Thermotoga *on plates, double strength (2 ×) of liquid SVO and various concentrations of agar or Gelrite were autoclaved separately and then mixed with equal volumes while they were still hot. The medium either was directly poured to Petri dishes for standard spreading or streaking, or was mixed with cell cultures prior to pouring for embedded growth. A Vacu-Quik jar (Almore International Inc., Portland, OR, USA) containing a packet of 4 g of palladium catalyst was used for anaerobic cultivation of plates. The atmosphere inside of the jar was exchanged to 96:4 N_2_-H_2 _before it was placed into an incubator of 77°C. Colonies usually appear in 24 h and grow bigger in 48 h. Kanamycin was supplemented when needed at 150 μg ml^-1 ^for liquid and 250 μg ml^-1 ^for soft and solid cultures. Cell growth in liquid was monitored by measuring the optical density of cell cultures at 600 nm (OD_600_). All aforementioned operations were carried out on the bench top.

**Table 1:** Strains & vectors used in this study

Strain or plasmid	Size (bp)	Description	Reference
***Thermotoga***			
*T*. *neapolitana *DSM 4359		Isolated from African continental solfataric springs	[25]
*T. maritima *MSB8		Isolated from geothermally heated sea floors in Italy and the Azores	[1]
*T*. sp. RQ7		Isolated from geothermally heated sea floors in Ribeira Quente, the Azores	[7]
***E. coli***			
DH5α		F^- ^*endA1 hsdR17 *(r_k_^-^, m_k_^+^) *supE44 thi-1 λ^- ^recA1 gyrA96 relA1 deoR *Δ(*lacZYA-argF*)- U169 ϕ80d*lacZ*ΔM15	[26]
**Plasmids**			
pUC19	2686	A high-copy number *E. coli *cloning vector containing portions of pBR322 and M13 mp19	GenBank: L09137
pRQ7	846	Cryptic miniplasmid from *T*. sp. RQ7	[5]
pKT1	3934	pUC-derived plasmid, containing a *kan *cassette for thermostable kanamycin selections	[13]
pDH1	3517	pRQ7 DNA cloned between BamHI and EcoRI sites of pUC19; Ap^r^	This study
pDH10	4762	pRQ7 DNA cloned between EcoRI and XbaI sites of pKT1; Ap^r^; Kan^r ^	This study; GenBank: JN813374

### Antibiotics sensitivity tests

One ml of overnight culture was mixed with 25 ml of hot SVO containing 0.3% Gelrite and poured into Petri dishes. Small discs of 7 mm in diameter were cut from Whatman qualitative filter paper and were placed on solidified plates. Various amount of kanamycin (50 ~ 250 μg) was added to the paper discs. After 48 h of anaerobic incubation, sensitive strains would display inhibition zones surrounding the discs. To specify the selective levels of the antibiotic in both liquid and solid media, kanamycin ranging from 50 to 300 μg ml^-1 ^was supplemented, and the proliferation of bacteria was monitored for up to 72 h.

### Extraction of DNA from *Thermotoga*

Plasmid DNA was extracted from *Thermotoga *using standard alkaline lysis method [11]. For genomic DNA, overnight culture of *Thermotoga *was extracted with equal volume of phenol: chloroform: isoamyl alcohol (volume ratio 25:24:1) followed by centrifugation at 13,523 *g *for 5 min to remove cell debris. DNA in the supernatant was precipitated with equal volume of isopropanol, washed once with 70% ethanol, air-dried, and dissolved in 10 mM Tris-EDTA buffer (pH 8.0) containing 20 μg ml^-1 ^RNase.

### Construction of pDH10

**Figure 1:**
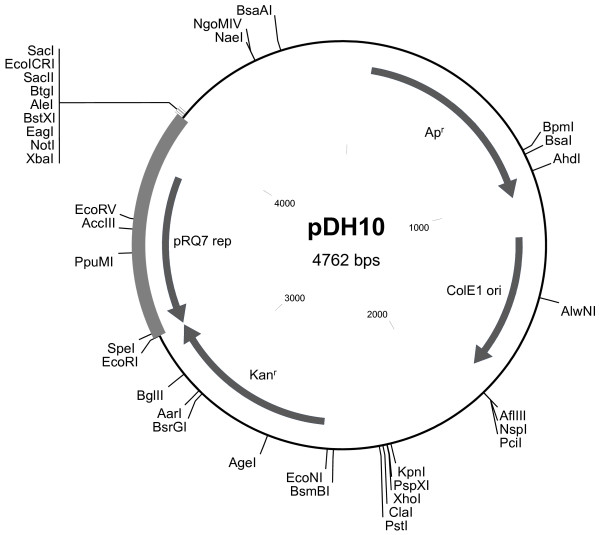
**Genetic map of the shuttle vector pDH10**. The region highlighted in bold represents the sequence of pRQ7. Enzymes with unique restriction sites are shown.

Plasmid pKT1 carrying a thermostable kanamycin adenyl transferase gene (*kan*) was purchased from Biotools, B & M Labs (Madrid, Spain). Primers 5'-GGGGATCCGAATGTGGTTAGTGTGATTAG-3' and 5'-GGGAATTCTTAACCATATCCCACTAGTTC-3' were used to amplify pRQ7, which was linearized immediately after the stop codon of the replication protein as the result of the PCR reactions. The PCR product was digested by BamHI and EcoRI and was inserted into the corresponding sites of pUC19 to give rise to pDH1. The XbaI-EcoRI fragment of pDH1 was then joined to pKT1 pre-digested with the same enzymes to generate pDH10 [GenBank:JN813374] (Figure 1). The constructs were confirmed by both restriction digestions and PCR. Plasmid pDH10 carries ColE1 origin of replication (*ori*) and β-lactamase (Ap^r^) for amplification and selection in *E. coli*, respectively. For its replication and selection in *Thermotoga*, pDH10 relies on the *ori *of pRQ7 and the engineered *kan *gene [12]. The expression of the *kan *gene is driven by a promoter from *Thermus thermophilus *HB8 [13], which is expected to be active in *Thermotoga *as well, since the consensus sequences of *Thermotoga *[14] and *Thermus *[15] promoters are nearly identical.

### Transformation and selection methods

Liposome-mediated transformation was conducted as previously described [9], except that all operations were carried out on the bench top. DOTAP liposomal reagent was purchased from Roche Diagnostics, Indianapolis, IN, USA. To prepare electrocompetent cells, overnight *Thermotoga *cultures were transferred to 50 ml of SVO liquid media and were allowed to grow until the cell density reached around 0.2. Cells were collected by centrifugation, washed once with cold deionized water and twice with the washing solution (10% glycerol, 0.85 M sucrose) and were resuspended in 500 μl of the same solution. For electroporation, 4 μg of plasmid DNA was mixed with 50 μl of the freshly made competent cells and incubated on ice for 5 minutes prior to introduction to a pre-chilled cuvette of 1 mm gap. The operation settings were 25 μF capacitance, 200 Ω resistance, and 1.5, 1.8, or 2 kV voltage (Gene Pulser Xcell™, Bio-Rad Laboratories, Hercules, CA). After electroporation, 1 ml of fresh SVO liquid medium was added to each cuvette, and the cell suspension was transferred to a N_2 _serum bottle and incubated at 77°C with gentle rotation for 3 h for recovery. Half of the recovered culture (500 μl) was then mixed with 25 ml of hot SVO solid medium supplemented with 250 μg ml^-1 ^kanamycin, poured to Petri dishes, and incubated in an anaerobic jar to retrieve transformants.

### Stability assays of the transformed DNA in *Thermotoga *and *E. coli*

Cultures of *Thermotoga *recombinant strains were transferred every 12 h for 3 days with an inoculum of 2% to a fresh SVO liquid medium in the presence or absence of kanamycin. With each transfer, an aliquot of the cultures was withdrawn and diluted to 10^-4^. Ten microliters of each diluted sample was then mixed with 10 ml of hot SVO solid medium with or without the antibiotic and was poured into a four-section Petri dish. In order to facilitate comparisons, samples of the same strain (but with different treatments) were arranged to different sections of the same plate. After incubation in an anaerobic jar for 48 h, colonies formed in each section were counted and compared.

To test the stability of pDH10 in *E. coli*, a liquid culture of DH5α/pDH10 was also transferred six times for every 12 h of growth in plain LB medium (no ampicillin) with 1% inoculum. Samples from each cycle were properly diluted and spread on plain LB plates to separate single colonies. One hundred such colonies were randomly chosen and were tested on LB plates containing 100 μg ml^-1 ^ampicillin. For control purposes, strain DH5α/pKT1 was tested in parallel.

## Results

### Improved methods for handling *Thermotoga *cultures in an aerobic environment

**Figure 2:**
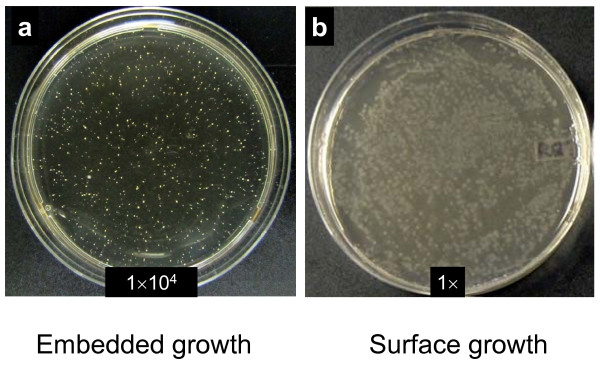
**Single colonies formed by *T*. sp. RQ7 cells**. (a) Embedded growth. Cells were mixed with hot SVO medium containing 0.3% Gelrite and were poured to Petri dishes before solidification. (b) Surface growth. Cells were spread evenly on the surface of freshly-made SVO plates containing 0.3% Gelrite and 0.7% agar. The number on each plate indicates the dilution factor of each culture.

The chance of obtaining *Thermotoga *transformants on plates can be seriously compromised if plating efficiencies are low. Considering that *Thermotoga *can tolerate brief exposures to oxygen, we simplified the overlay methods used by other groups (Kenneth Noll, University of Connecticut, private communication; [16]) and developed an embedded growth method. Properly diluted liquid cultures were suspended in hot SVO containing 0.3% Gelrite, and the mixtures were allowed to solidify in Petri dishes. In this method, cells were embedded in the medium matrix, and their exposure to oxygen was reduced. Ten microliters of an overnight culture of *T*. sp. RQ7 with a dilution factor of 10^4 ^formed 1256 colonies (Figure 2a), which equals to 1.26 × 10^9 ^colony forming units (CFU) per ml. By contrast, a surface culture, prepared by standard spreading in the same environment, would typically generate 7.56 × 10^3 ^CFU ml^-1 ^(Figure 2b), about ten thousand times less. Given that *T*. *neapolitana *cultures contain approximate 3.0 × 10^9 ^cells after growing in liquid SVO for 14 h at 70°C [17], we estimate that the plating efficiency of our embedded method is close to 50%. This high efficiency enables us to select or screen a large number of single colonies while still enjoying the convenience of aerobic handling.

To facilitate the transfer of single colonies from solid to liquid media under aerobic conditions, we introduced a soft SVO medium by adding 0.075% Gelrite to liquid SVO. Gelrite prevents atmospheric oxygen from penetrating deep into the medium. To transfer cultures from solid to soft SVO, single colonies were picked up from plates by a loop and were pushed down to the bottoms of the test tubes containing soft SVO, where a local anaerobic environment has been created. After 12-24 h of incubation, cultures grown in soft SVO were then transferred to liquid SVO by a syringe. Although the introduction of soft SVO seemed to prolong the overall operation cycle, it ensured maximum viability of *Thermotoga *cells during the transfer, which eventually allowed us to isolate *Thermotoga *transformants for the first time (see below). Soft SVO may also serve as an excellent storage medium for *Thermotoga*. Cultures kept at the bench top for 2 months were still vital and exhibited no growth defects.

### Kanamycin is a suitable selection marker for *T*. sp. RQ7 and *T. maritima*

**Figure 3:**
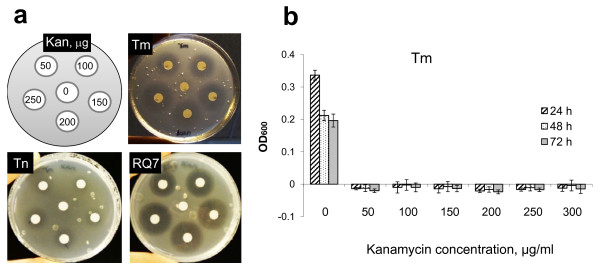
**Sensitivity of *Thermotoga *spp. to kanamycin**. (a) Sensitive cells formed inhibition zones surrounding the paper discs loaded with various amounts of the antibiotic, as indicated in the top left panel. Gas bubbles produced by the *Thermotoga *cells were clearly visible in each plate. (b) Optical densities of *T. maritima *liquid cultures grown with kanamycin ranging from 0 to 300 μg/ml. Results of three independent tests. Tm, *T. maritima*; Tn, *T. neapolitana*; RQ7, *T*. sp. RQ7.

To determine whether kanamycin is a suitable selection marker for *Thermotoga*, one needs to know the sensitivity of *Thermotoga *host strains. An initial study indicates that *T. maritima *is sensitive to kanamycin [9], but a more recent work states that it is highly resistant to the antibiotic [16]. For *T*. sp. RQ7, there are simply no related reports. We decided to clarify the discrepancy of the previous findings on *T. maritima *and to determine the sensitivity of *T*. sp. RQ7. Small discs of filter paper loaded with various amount of kanamycin were mounted on top of the SVO plates premixed with *Thermotoga *cultures (Figure 3a). After two days of incubation, cells not affected by the antibiotic grew into a dense lawn, forming a visible background. Sensitive cells in close proximity to the paper discs were unable to grow, resulting in clear halos. The inhibition zones formed on *T. maritima *plates (Figure 3a) revealed that this strain is indeed sensitive to kanamycin. A distinctive zone was visible even with the lowest concentration of kanamycin. *T*. sp. RQ7 displayed a similar level of sensitivity to the drug. As for *T*. *neapolitana*, slight inhibition was noticed when 100 μg of kanamycin was used, and a small inhibition zone was only apparent when 250 μg of the drug was used. Therefore, kanamycin may serve as a good selection marker for *T*. sp. RQ7 and *T. maritima*, but not for *T*. *neapolitana*.

We next specified the selective levels of kanamycin in both liquid and solid media. The growth of *T. maritima *in liquid SVO was completely inhibited by 50 μg ml^-1 ^kanamycin for at least 72 h (Figure 3b). However, spontaneous mutations sometimes caused the cultures to become resistant to the antibiotic, and a complete inhibition over a period of 72 h was only possible when the input amount was increased to 150 μg ml^-1^. Similar phenomena were also noticed with *T*. sp. RQ7. On SVO plates, spontaneous mutants of *T. maritima *and *T*. sp. RQ7 occasionally appeared after 48 h of incubation when up to 200 μg ml^-1 ^kanamycin was added, but they rarely appeared when the antibiotic concentration was increased to 250 μg ml^-1^. Based on these observations, for the rest of the study, kanamycin was added at 150 μg ml^-1 ^to liquid media and at 250 μg ml^-1 ^to soft and solid media.

### Transformation of *Thermotoga-E. coli *shuttle vector pDH10

Because most bacteria become competent after a short electric pulse, electroporation was attempted to introduce pDH10 to *Thermotoga*. Electric pulses of various strengths were applied to both *T*. sp. RQ7 and *T. maritima *in the presence of 4 μg of plasmid DNA, and the transformants were selected with embedded growth. When an electric pulse of 2.0 kV was employed, five *T*. sp. RQ7 and one *T. maritima *transformants were obtained. A pulse of 1.8 kV resulted in eight *T*. sp. RQ7 and no *T. maritima *transformants. When the voltage dropped to 1.5 kV, no transformants were available with either species. These results suggest that the optimal voltage for *Thermotoga *is around 1.8 to 2.0 kV. In the control experiment, *T*. sp. RQ7 and *T. maritima *cells were treated with a pulse of 1.8 kV in the absence of DNA, and no spontaneous mutants were found.

**Figure 4:**
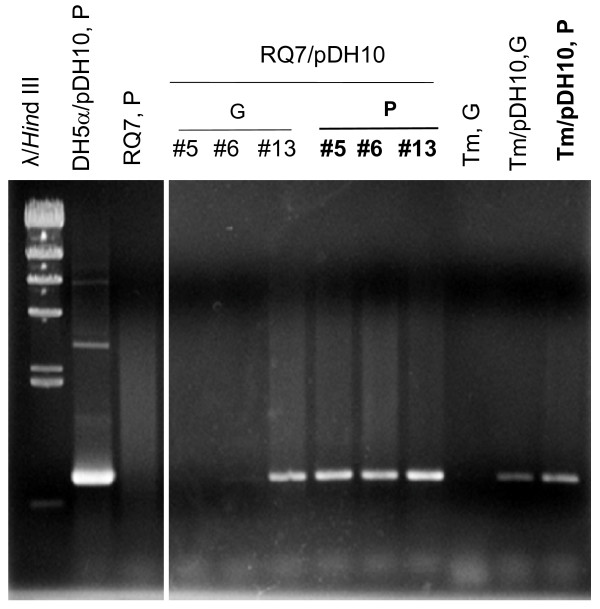
**Detection of the transformed *kan *gene**. PCR products of the *kan *gene were obtained from the plasmid extracts (P, labeled in bold) or the genomic DNA preparations (G) of the recombinant strains. Three RQ7/pDH10 and one Tm/pDH10 transformants, all obtained by electroporation, were examined. Plasmid extracts from DH5α/pDH10 and *T*. sp. RQ7 were included as positive and negative controls, respectively. Analyzed with a 0.8% agarose gel.

**Figure 5:**
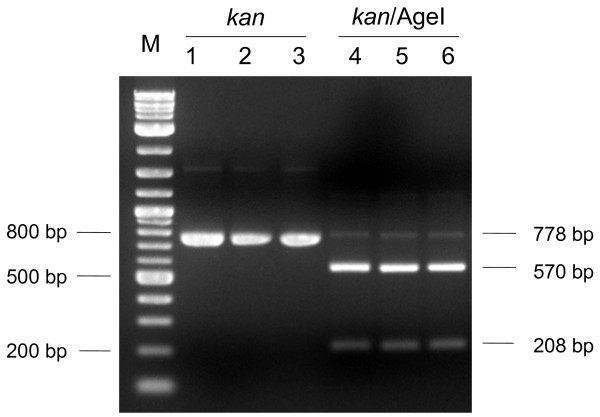
**Restriction digestions of the *kan *gene**. PCR products of the *kan *gene were prepared from the plasmid extracts of DH5a/pDH10 (lanes 1 and 4), RQ7/pDH10 (lanes 2 and 5), and Tm/pDH10 (lanes 3 and 6). Lanes 1-3 represent the PCR products of the *kan *gene, and lanes 4-6 are samples digested by AgeI. M, 2-log DNA ladder. Analyzed with a 2% agarose gel.

All transformants (designated as RQ7/pDH10 or Tm/pDH10 hereafter) displayed visible growth after a 24 h incubation in soft SVO. Three RQ7/pDH10 strains (#5, #6, and #13) and the single Tm/pDH10 strain were propagated in liquid SVO for extraction of plasmid and genomic DNA. On agarose gels, no pDH10 DNA could be detected from the plasmid extract of any strain, even though pRQ7 was clearly visible from the three RQ7/pDH10 samples, indicating that the extraction procedure was successful. Plasmid and genomic DNA extracted from an equal amount of each transformant culture was then subject to PCR analysis. A fragment of 778 bp, corresponding to the size of the *kan *gene, was obtained from each plasmid extract (lanes are labeled in bold in Figure 4) but was missing from the genomic DNA of RQ7/pDH10 #5 and #6. The PCR products were gel-purified and were subject to restriction digestion with AgeI, which is expected to cleave the *kan *gene into two fragments of 208 and 570 bp. Indeed, the digestion reactions released these expected fragments from every sample (Figure 5), indicating that the PCR products were authentic *kan *genes.

For validation and comparison purposes, pDH10 was also introduced to *T*. sp. RQ7 and *T. maritima *through liposome-mediated transformation. Four *T*. sp. RQ7 and five *T. maritima *transformants were obtained from 1 μg of plasmid DNA, as opposed to zero colonies from the samples treated with liposomes containing no DNA. All transformants grew well in both soft and liquid selective media, and the presence of the *kan *gene was also confirmed by PCR.

### Transformed DNA was stably maintained in *Thermotoga*

To determine the stability of the transformed DNA, liquid cultures of RQ7/pDH10 and Tm/pDH10 were transferred every 12 h, for six consecutive times, to fresh media in the presence or absence of kanamycin. Cultures from each transfer cycle were tested with both SVO (no kanamycin) and SVO+Kan plates. The number of colonies on a plain SVO plate represents the quantity of total viable cells in a sample, whereas the number from a SVO+Kan plate defines the abundance of resistant cells. Surprisingly, by the end of the experiment, ~100% of both RQ7/pDH10 and Tm/pDH10 still had the transformed DNA, even without the selective pressure (Table 2). The *kan *gene was confirmed by PCR from the plasmid preparations of all strains after each transfer.

**Table 2:** Percentage of *Thermotoga *colonies resistant to kanamycin after six transfers*

Recombinant strain	Transfer medium	Resistant colonies (%)
RQ7/pDH10	SVO	104.92 ± 9.57
	SVO + Kan	106.66 ± 3.56
Tm/pDH10	SVO	100.96 ± 23.72
	SVO + Kan	108.59 ± 7.54

*Results of three independent tests.

### Incorporation of pRQ7 increased the stability of pUC19 derivatives in *E. coli*

**Figure 6:**
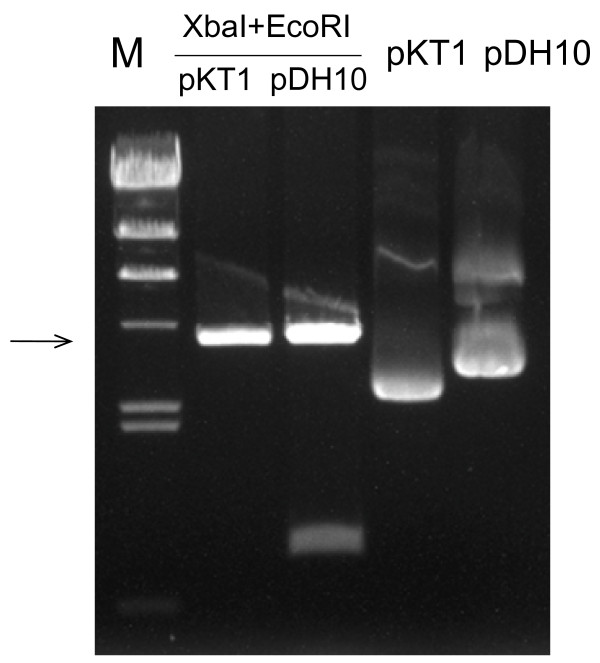
**Comparison of the copy numbers of pDH10 and pKT1 in *E. coli***. Plasmid DNA was extracted from the same amount of recombinant cells and was digested by XbaI and EcoRI. The arrow indicates the shared sequence of the two vectors. Analyzed with a 0.8% agarose gel.

The stable maintenance of the transformed DNA in *Thermotoga *motivated us to test the stability of pDH10 in *E. coli *under non-selective conditions. The parent vector pKT1, a derivative of pUC19, was used as the control. Interestingly, pDH10 was much more stable in *E. coli *than pKT1 was. The parent vector pKT1 was eliminated by ~90% of the host cells after a single transfer and was completely lost from the population after three transfers (Table 3). By contrast, pDH10 was eliminated at a much slower rate. It was carried by ~90% of the cells after three transfers and ~32% of the cells after six transfers. Intact pDH10 was obtained from the resistant colonies. It is noteworthy to mention that similar results were also obtained when pDH1 and pUC19 were compared in the same way. These data demonstrate that the insertion of the pRQ7 sequence somehow enhances the stability of pUC family vectors. We next compared the copy numbers of pDH10 and pKT1 in their *E. coli *hosts. The two vectors were prepared from the same amount of cells and were digested by XbaI and EcoRI. These double digestions released the pRQ7 sequence from pDH10 (Figure 1 &6). The abundances of the shared vector backbone of pDH10 and pKT1 were comparable on an agarose gel (indicated by the arrow in Figure 6), suggesting that the copy numbers of two vectors were similar. Therefore, the dramatically improved stability of pDH10 is not caused by an increase in copy number.

**Table 3:** Percentage of *E. coli *colonies resistant to ampicillin during consecutive transfers*

Number of transfers	pDH10	pKT1
1	96.75 ± 1.30	7.25 ± 5.54
2	95.5 ± 2.50	1.75 ± 1.48
3	90 ± 6.04	0
4	68.5 ± 6.34	-
5	44 ± 11.68	-
6	32.25 ± 15.20	-

*Results of four independent tests.

## Discussion

### Improved method for cultivation of *Thermotoga*

The success of isolating transformants from solid media is essential to any genetic manipulation attempt. Whereas this is not a concern with aerobic mesophiles like *E. coli*, this requirement has become a limiting factor for the genetic investigations of many strict anaerobic, hyperthermophilic organisms. One obstacle is the requirement of an anaerobic glove box to handle plates. Since picking up colonies requires great precision, reaching out to a single colony with an inoculation loop or a toothpick through thick gloves has proven to be challenging for many of us. Even though gloveless chambers are commercially available, they are costly to maintain. Rolling tubes or tissue culture flasks in combination to Hungate techniques may serve as alternatives [16,18-20], but they are prone to cross contaminations due to the narrow openings of these containers. Based on the fact that *Thermotoga *are fairly oxygen-tolerant, especially when they are not actively growing [1,17,21,22], we prepare *Thermotoga *solid cultures with an embedded method, independent of an anaerobic chamber or an anoxic gas conduit. Our method sustains ~50% plating efficiency, making it possible to select for *Thermotoga *transformants among a sizeable population of viable cells. In addition, we developed a soft SVO medium to bridge the transfer of cultures from solid media to liquid media in an aerobic environment. Soft SVO is easy to make and convenient to use, and it also allows the withdrawal of cultures using a syringe.

### Transformation and expression of the *kan *gene

A heterologous *kan *gene has been functionally expressed and stably established in *Thermotoga*. The *kan *gene, carried on the shuttle vector pDH10, was introduced to *Thermotoga *by both electroporation and liposome-mediated transformation. The latter approach yields higher transformation efficiency (4~5 transformants per μg of DNA), but the former has the potential to mass-produce competent cells for future needs. Electrocompetency has been established in *Thermotoga *in this study, and a transformation efficiency of ~2 transformants per μg of DNA has been observed in RQ7 and ~0.25 in *T. maritima*. Two factors might have caused the transformation efficiencies to be low: the damages caused by oxygen and the activities of restriction-modification systems. Because the preparation of electrocompetent cells and the transformation procedure were performed under aerobic conditions, a significant portion of the *Thermotoga *cells would not survive the handling. Performing the experiment in an anaerobic chamber should help to improve the transformation efficiencies, albeit the operations would be cumbersome. In addition, restriction-modification systems have been discovered in *Thermotoga*, for instance, we have recently characterized a *Thermotoga*-specific Type II restriction-modification system [23]. Unmethylated foreign DNA will be restricted by host-specific restriction nucleases as soon as they enter *Thermotoga *cells. Proper methylation of pDH10 prior to transformation is expected to significantly increase the quantity of transformants. Indeed, electrotransformation efficiency in *Clostridia *has been improved by 10^4^~10^6 ^folds by methylating the shuttle vectors [24].

Although intact plasmid DNA of pDH10 has not been detected in *Thermotoga*, we favor the idea that pDH10 is autonomous in *Thermotoga*, because the *kan *gene is always associated to a plasmid preparation instead of a genomic DNA extract (Figure 4). If pDH10 had been integrated into the chromosome, we would have seen the *kan *gene appearing more often from the genomic samples than from the plasmid extracts. Our data stated otherwise (Figure 4), suggesting that pDH10 is likely an independent molecule rather than part of the chromosome. Because genomic DNA can include both chromosomal and plasmid DNA, it is not surprising to occasionally obtain a positive signal from a genomic DNA sample, even though the *kan *gene is carried by a free-living plasmid. We suspect that pDH10 has an extremely low copy number in *Thermotoga*, because the attempts to detect it by inverse PCR (amplifying pDH10 outward from the *kan *gene), retransformation (transform *E. coli *with plasmid extracts of *Thermotoga *transformants), or Southern blotting (using digoxygenin-labeled probes made from either the *kan *gene or pRQ7) did not generate any signals. The expected inverse PCR product is ~4 kb, much bigger than the *kan *gene. This makes the inverse PCR technically more challenging than just amplifying the *kan *gene.

## Conclusions

In spite of the intriguing questions awaited to be answered with these elusive organisms, the door to the genetic manipulation of *Thermotoga *has been reopened. Ten years after the report of transient expression of heterologous genes in *Thermotoga *[9], we confirm that *Thermotoga *are truly transformable, not only by liposome-mediated transformation, but also by electroporation. Engineered *Thermotoga *strains are available for the first time.

## List of abbreviations

Ap: ampicillin; CFU: colony forming unit; DNA: deoxyribonucleic acid; EDTA: ethylenediaminetetraacetic acid; Kan: kanamycin; LB: Luria Broth; PCR: polymerase chain reaction; Tm: *Thermotoga maritima*; Tn: *Thermotoga neapolitana*; RQ7; *Thermotoga *sp. RQ7.

## Competing interests

DH and ZX have one or more pending patent applications on the described technology.

## Authors' contributions

ZX conceived and coordinated the study and drafted the manuscript. ZX and DH designed the experiments and analyzed the data. DH carried out the experiments. SMN contributed to the stability study of pDH10 in *E. coli*. All authors read and approved the final manuscript.

## References

[B1] Huber R Langworthy TA Konig H Thomm M Woese CR Sleytr UB Stetter KO Thermotoga maritima sp. nov. represents a new genus of unique extremely thermophilic eubacteria growing up to 90 degrees C Archives of Microbiology 1986 144 4 324 333 10.1007/BF00409880

[B2] Windberger E Huber R Trincone A Fricke H Stetter KO Thermotoga thermarum sp. nov. and Thermotoga neapolitana occurring in African continental solfataric springs Archives of Microbiology 1989 151 6 506 512 10.1007/BF00454866

[B3] Takahata Y Nishijima M Hoaki T Maruyama T Thermotoga petrophila sp. nov. and Thermotoga naphthophila sp. nov., two hyperthermophilic bacteria from the Kubiki oil reservoir in Niigata, Japan International Journal of Systematic and Evolutionary Microbiology 2001 51 1901 1909 10.1099/00207713-51-5-1901 11594624

[B4] Schroder C Selig M Schonheit P Glucose Fermentation to Acetate, CO2 and H2 in the Anaerobic Hyperthermophilic Eubacterium Thermotoga-Maritima - Involvement of the Embden-Meyerhof Pathway Archives of Microbiology 1994 161 6 460 470

[B5] Harriott OT Huber R Stetter KO Betts PW Noll KM A Cryptic Miniplasmid from the Hyperthermophilic Bacterium Thermotoga Sp Strain Rq7 Journal of bacteriology 1994 176 9 2759 2762 8169230 10.1128/jb.176.9.2759-2762.1994 PMC205421

[B6] Akimkina T Ivanov P Kostrov S Sokolova T Bonch-Osmolovskaya E Firman K Dutta CF McClellan JA A highly conserved plasmid from the extreme thermophile Thermotoga maritima MC24 is a member of a family of plasmids distributed worldwide Plasmid 1999 42 3 236 240 10.1006/plas.1999.1429 10545265

[B7] Nesbo CL Dlutek M Doolittle WF Recombination in thermotoga: Implications for species concepts and biogeography Genetics 2006 172 2 759 769 16322518 10.1534/genetics.105.049312 PMC1456242

[B8] Yu JS Noll KM Plasmid pRQ7 from the hyperthermophilic bacterium Thermotoga species strain RQ7 replicates by the rolling-circle mechanism Journal of bacteriology 1997 179 22 7161 7164 9371465 10.1128/jb.179.22.7161-7164.1997 PMC179659

[B9] Yu JS Vargas M Mityas C Noll KM Liposome-mediated DNA uptake and transient expression in Thermotoga Extremophiles 2001 5 1 53 60 10.1007/s007920000173 11302503

[B10] Van Ooteghem SA Beer SK Yue PC Hydrogen production by the thermophilic bacterium Thermotoga neapolitana Applied Biochemistry and Biotechnology 2002 98 177 189 10.1385/ABAB:98-100:1-9:177 12018246

[B11] Sambrook J Russell DW The condensed protocols from molecular cloning: a laboratory manual 2006 Cold Spring Harbor Laboratory Press

[B12] Liao H McKenzie T Hageman R Isolation of a thermostable enzyme variant by cloning and selection in a thermophile Proceedings of the National Academy of Sciences of the United States of America 1986 83 3 576 580 10.1073/pnas.83.3.576 3003740 PMC322906

[B13] Lasa I Caston JR Fernandez-Herrero LA de Pedro MA Berenguer J Insertional mutagenesis in the extreme thermophilic eubacteria Thermus thermophilus HB8 Molecular microbiology 1992 6 11 1555 1564 10.1111/j.1365-2958.1992.tb00877.x 1625584

[B14] Liao D Dennis PP The organization and expression of essential transcription translation component genes in the extremely thermophilic eubacterium Thermotoga maritima The Journal of biological chemistry 1992 267 32 22787 22797 1429627

[B15] Maseda H Hoshino T Screening and analysis of DNA fragments that show promoter activities in Thermus thermophilus FEMS Microbiol Lett 1995 128 2 127 134 10.1111/j.1574-6968.1995.tb07511.x 7750730

[B16] Jiang Y Zhou Q Wu K Li XQ Shao WL A highly efficient method for liquid and solid cultivation of the anaerobic hyperthermophilic eubacterium Thermotoga maritima Fems Microbiology Letters 2006 259 2 254 259 10.1111/j.1574-6968.2006.00273.x 16734788

[B17] Van Ooteghem SA Jones A Van Der Lelie D Dong B Mahajan D H(2) production and carbon utilization by Thermotoga neapolitana under anaerobic and microaerobic growth conditions Biotechnol Lett 2004 26 15 1223 1232 15289678 10.1023/B:BILE.0000036602.75427.88

[B18] Hermann M Noll KM Wolfe RS Improved Agar Bottle Plate for Isolation of Methanogens or Other Anaerobes in a Defined Gas Atmosphere Appl Environ Microbiol 1986 51 5 1124 1126 16347059 10.1128/aem.51.5.1124-1126.1986 PMC239021

[B19] Macy JM Snellen JE Hungate RE Use of syringe methods for anaerobiosis The American journal of clinical nutrition 1972 25 12 1318 1323 4565348 10.1093/ajcn/25.12.1318

[B20] Miller TL Wolin MJ A serum bottle modification of the Hungate technique for cultivating obligate anaerobes Applied microbiology 1974 27 5 985 987 4598231 10.1128/am.27.5.985-987.1974 PMC380188

[B21] Childers SE Vargas M Noll KM Improved Methods for Cultivation of the Extremely Thermophilic Bacterium Thermotoga-Neapolitana Applied and Environmental Microbiology 1992 58 12 3949 3953 16348824 10.1128/aem.58.12.3949-3953.1992 PMC183209

[B22] Le Fourn C Fardeau ML Ollivier B Lojou E Dolla A The hyperthermophilic anaerobe Thermotoga Maritima is able to cope with limited amount of oxygen: insights into its defence strategies Environmental microbiology 2008 10 7 1877 1887 10.1111/j.1462-2920.2008.01610.x 18397308

[B23] Xu Z Han D Cao J Saini U Cloning and characterization of the TneDI restriction: modification system of Thermotoga neapolitana Extremophiles 2011 15 6 665 672 10.1007/s00792-011-0397-9 21918796

[B24] Mermelstein LD Papoutsakis ET In vivo methylation in Escherichia coli by the Bacillus subtilis phage phi 3T I methyltransferase to protect plasmids from restriction upon transformation of Clostridium acetobutylicum ATCC 824 Appl Environ Microbiol 1993 59 4 1077 1081 8386500 10.1128/aem.59.4.1077-1081.1993 PMC202241

[B25] Belkin S Wirsen CO Jannasch HW A New Sulfur-Reducing, Extremely Thermophilic Eubacterium from a Submarine Thermal Vent Applied and Environmental Microbiology 1986 51 6 1180 1185 16347075 10.1128/aem.51.6.1180-1185.1986 PMC239042

[B26] Grant SG Jessee J Bloom FR Hanahan D Differential plasmid rescue from transgenic mouse DNAs into Escherichia coli methylation-restriction mutants Proceedings of the National Academy of Sciences of the United States of America 1990 87 12 4645 4649 10.1073/pnas.87.12.4645 2162051 PMC54173

